# Non-operative Management of Locally Advanced Rectal Adenocarcinoma Using a Watch-and-Wait Approach: A Report of Two Cases

**DOI:** 10.7759/cureus.87522

**Published:** 2025-07-08

**Authors:** Elene Zaalishvili, Irine Khubua

**Affiliations:** 1 Internal Medicine, David Tvildiani Medical University, Tbilisi, GEO; 2 Clinical Oncology, Caucasus Medical Centre, Tbilisi, GEO

**Keywords:** complete clinical response, non-operative management, rectal adenocarcinoma, t3n1m0 rectal cancer, total neoadjuvant therapy

## Abstract

The rectum constitutes the distal segment of the large intestine, and rectal cancer typically arises through a multistep process in which normal epithelial cells undergo dysplastic transformation, eventually progressing to invasive carcinoma. This progression is often asymptomatic and may take years, making early detection challenging. Consequently, many cases are diagnosed at advanced stages. Management strategies are highly dependent on the stage at diagnosis. For T3N1M0 rectal cancers, current guidelines recommend neoadjuvant chemoradiotherapy followed by surgical resection. However, when the tumor location precludes sphincter-sparing surgery, patients may require a permanent colostomy, significantly affecting quality of life.

We present two cases of rectal adenocarcinoma in which patients underwent total neoadjuvant therapy, achieved a complete clinical response, and opted for non-operative management with active surveillance. These cases illustrate the potential role of a non-surgical, observation-based approach in selected patients and emphasize the importance of shared decision-making and individualized treatment planning in rectal cancer care.

## Introduction

The rectum comprises the final six inches of the large intestine. Rectal cancer develops when cells in this region begin to grow uncontrollably. Several risk factors contribute to this, including a diet high in processed meats and low in fiber and vegetables, smoking, alcohol consumption, obesity, and increasing age [1]. The average age at diagnosis is typically in the sixth decade of life. However, this pattern is changing.

In the United States alone, approximately 46,000 new cases of rectal cancer are diagnosed annually, with about 95% being adenocarcinomas [1,2]. While the incidence of rectal cancer is declining in older adults, thanks to effective screening programs, it is rising among younger individuals. This increase is especially pronounced in those under 50 years of age [3], where the incidence of colorectal cancer (CRC) rose from 8.6 per 100,000 in 1992 to 12.9 per 100,000 in 2018 [4]. This shift presents a growing concern for physicians. Treating a patient in their 30s or 40s means not only aiming to prolong life but also preserving a quality of life suitable for a younger person. This raises important questions about the optimal management of rectal cancer in younger patients. While evidence-based guidelines exist, are there situations where it is appropriate to deviate from them in favor of a more individualized approach? This is the central question our case report seeks to explore. 

Management strategies for rectal cancer depend largely on the stage at diagnosis. Our focus is on stage T3N1M0 rectal cancer. According to current guidelines, patients with resectable T3N1 tumors and no distant metastases have several treatment options: primary surgery, preoperative chemoradiotherapy (CRT) with fluoropyrimidine-based regimens over 5.5 to 6 weeks, or a short-course high-dose preoperative radiotherapy over five days, often referred to as the "Swedish style” [5-7]. Postoperatively, these patients typically receive at least four months of adjuvant chemotherapy. All of these approaches involve surgical intervention.

But what if a patient shows a complete clinical response (cCR) after neoadjuvant chemoradiotherapy - treatment given before surgery to shrink the tumor - and is not a candidate for sphincter-sparing surgery? In such cases, a non-operative "watch-and-wait" approach, involving close surveillance rather than immediate resection, may be considered. This remains an area of active debate. While many physicians advocate for immediate surgical resection, it’s important to consider the substantial impact on quality of life following abdominoperineal resection (APR), particularly in younger patients. 

Our case report presents two such patients to shed light on this complex issue. By highlighting these scenarios, we aim to increase awareness and encourage further study of alternative management strategies. With more data and open discussion, it may become more acceptable, and even preferable, for clinicians to consider non-traditional approaches that align better with the needs and values of the patients.

## Case presentation

Case 1

**Figure 1:**
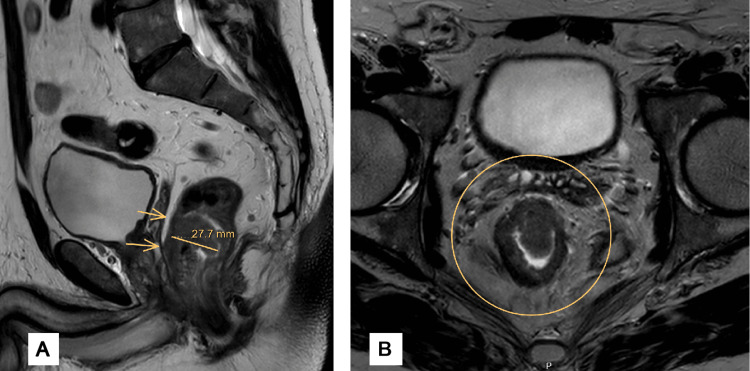
Baseline pelvic MRI obtained before initiation of total neoadjuvant chemoradiotherapy. A. Sagittal T2-weighted pelvic MRI demonstrating a rectal wall mass with a maximum thickness of 27.7 mm (indicated by arrows). The lesion is closely approximated to the posterior aspect of the prostate; however, a preserved intervening plane of perirectal fat is evident, suggesting the absence of definitive anterior organ invasion. B. Axial T2-weighted pelvic MRI demonstrating loss of the normal trilaminar architecture of the rectal wall. There is stranding and disruption of the surrounding mesorectal fat, indicative of extramural tumor extension.

**Figure 2:**
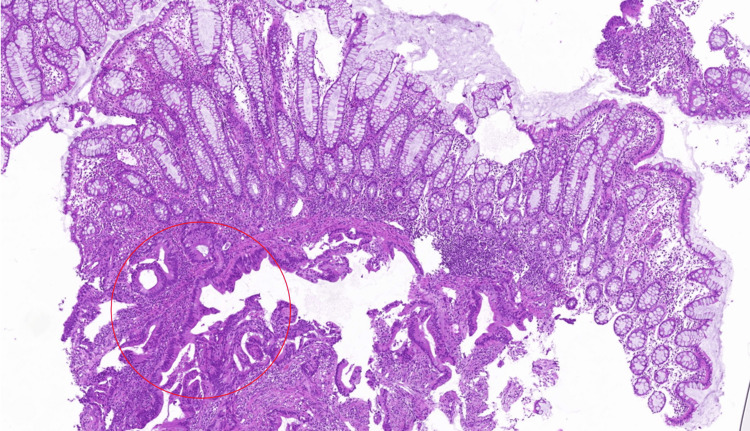
Rectal mucosal biopsy stained with hematoxylin and eosin (H&E) showing transition from normal colonic glands to infiltrative atypical glands (circled) consistent with adenocarcinoma (10x magnification).

A 53-year-old man presented in February 2021 with several months of constipation, generalized fatigue, mucous stools, and unintentional weight loss. Pelvic MRI (February 28, 2021) revealed abnormal thickening of the rectal wall, 25 mm from the anal verge (Figure 1). The mass was adherent to the prostate and levator ani muscles, with obliteration of the surrounding fat planes. No evidence of extramural vascular invasion (EMVI) was identified on MRI. Pararectal lymph nodes with irregular margins were noted, the largest measuring 6 mm. An abdominal CT performed in the same period confirmed an irregular, circumferential thickening of the rectal wall (~2 cm) over a 7 cm segment. The rectosigmoid junction was not involved. The lesion had infiltrated the muscularis propria, as well as the pararectal and presacral fat. Baseline and serial carcinoembryonic antigen (CEA) levels during treatment remained within the normal range, and the cancer was therefore assessed as marker-insensitive with respect to CEA. Biopsy confirmed moderately differentiated rectal adenocarcinoma (Figure 2). Based on the combined imaging and histopathological findings, the tumor was staged as T3N1M0.

The patient began neoadjuvant chemotherapy with the XELOX regimen (capecitabine and oxaliplatin) in March 2021. A follow-up MRI in May 2021 demonstrated significant tumor regression, and the regimen was continued. From June 26 to August 4, 2021, the patient received concurrent radiotherapy, with a total dose of 45 gray to the pelvic lymph nodes and rectum, followed by a 5.4 gray boost to the rectal adenocarcinoma. Subsequent imaging demonstrated a complete radiological response. At that time, endoscopic assessment demonstrated a complete response, characterized by mucosal whitening and scar formation at the site of the previous tumor, with no evidence of ulceration, mass, or mucosal irregularities.

**Figure 3:**
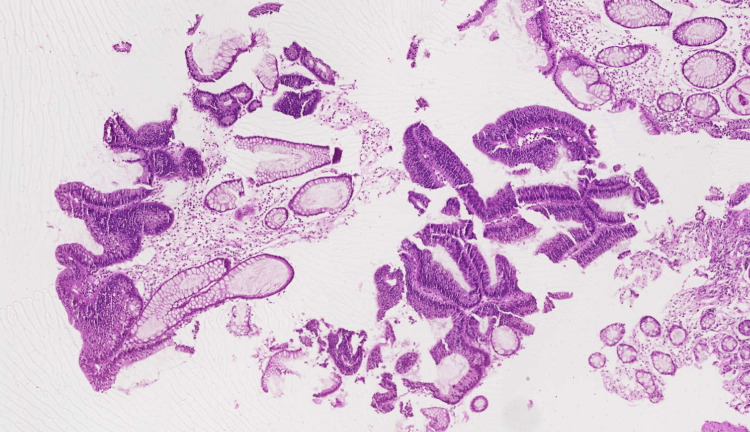
Hematoxylin and eosin (H&E)-stained rectal mucosal biopsy demonstrating a villous adenoma at 10x magnification.

The patient expressed a preference to avoid surgical resection, and non-operative management (NOM) with active surveillance was chosen through shared decision-making. In December 2023, a surveillance colonoscopy revealed an irregular lesion on the posterior rectal wall, 2-3 cm from the anal verge, without evidence of luminal narrowing. Biopsy confirmed a villous adenoma with high-grade dysplasia, which was successfully managed endoscopically (Figure 3).

**Figure 4:**
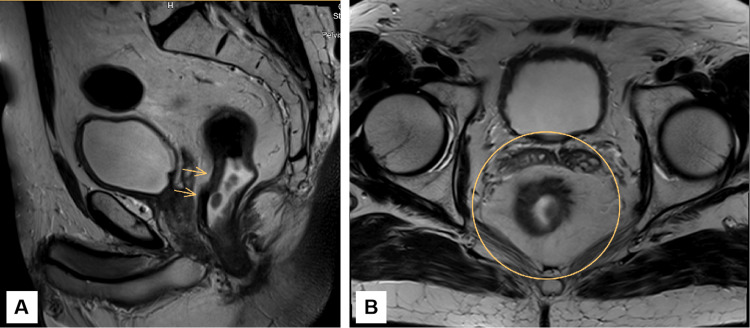
Latest pelvic MRI demonstrating post-treatment findings after total chemoradiotherapy. A. Sagittal T2-weighted pelvic MRI demonstrating wall thickening in the mid and lower rectum. There is hypointense fibrosis within the rectal wall, consistent with post-treatment changes. The mesorectal fat remains preserved, with no evidence of extramural invasion. The site of the primary tumor (indicated by arrows) shows features of tumor regression and fibrosis. B. Axial T2-weighted pelvic MRI showing hypointense concentric thickening of the rectal wall, suggestive of fibrosis. No definitive intermediate signal intensity mass is identified. There are no grossly enlarged mesorectal lymph nodes with suspicious features.

As of February 2025, follow-up imaging remains stable with no evidence of recurrence (Figure 4). The patient is asymptomatic and has been following a regular surveillance schedule, which included endoscopy and pelvic MRI every four months during the first two years, followed by every six months thereafter. Chest and abdominal CT scans were performed every six months for the first two years, then annually.

Case 2

**Figure 5:**
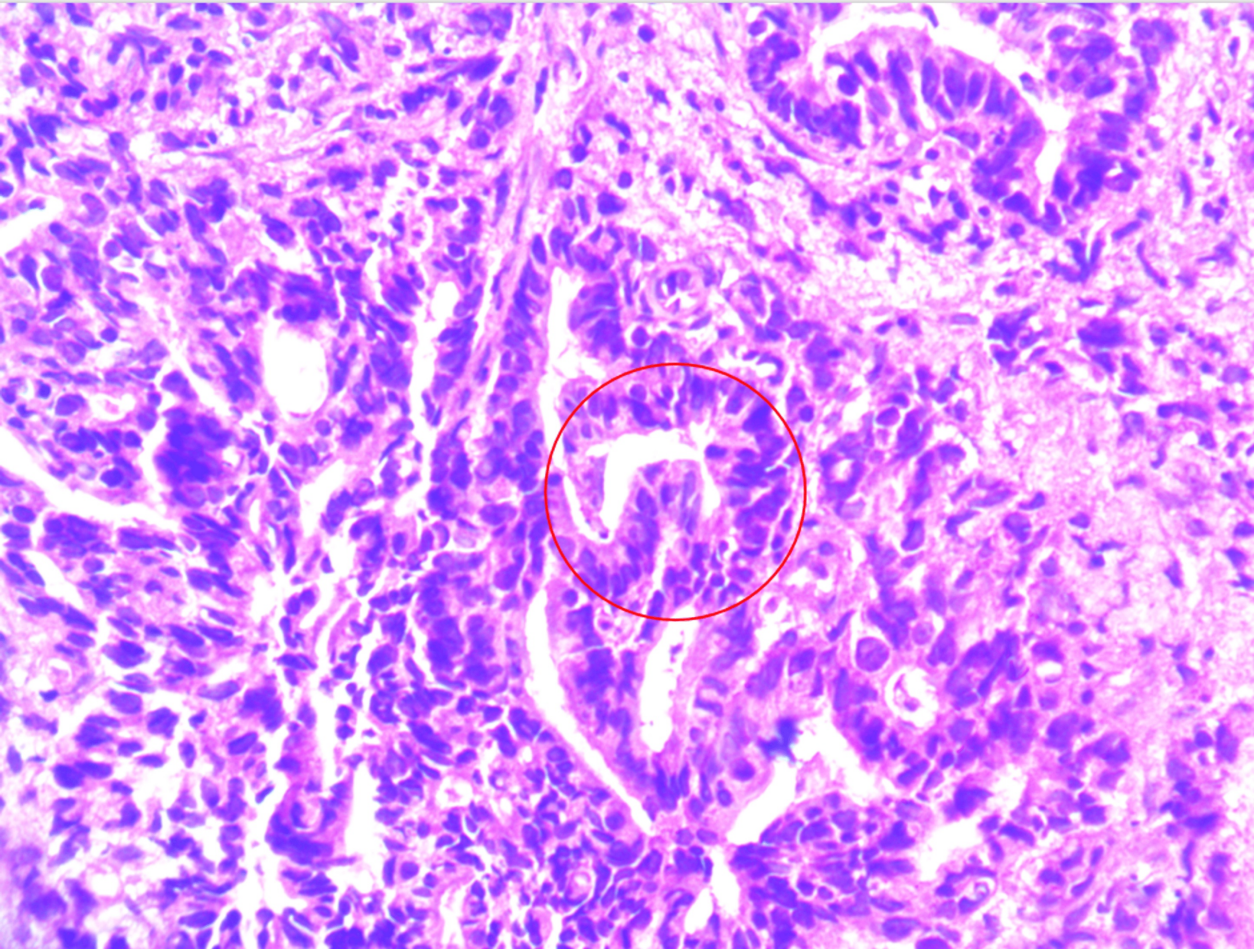
Hematoxylin and eosin (H&E)-stained section of rectal mucosa showing features of rectal adenocarcinoma, including glandular formation and nuclear atypia (circled) (high power).

**Figure 6:**
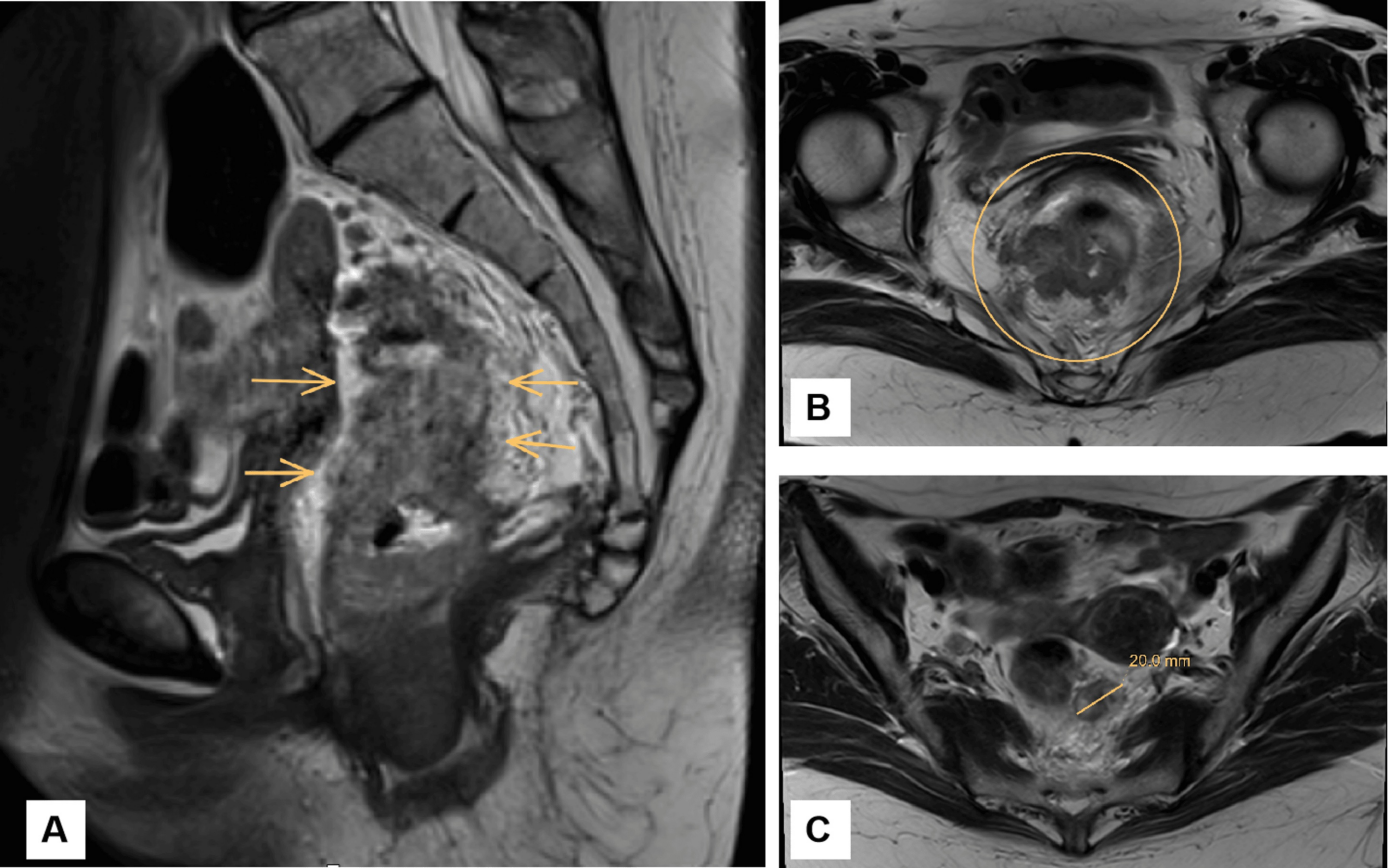
Baseline pelvic MRI obtained before initiation of total neoadjuvant chemoradiotherapy. A. Sagittal T2-weighted pelvic MRI demonstrating marked thickening and irregularity of the rectal wall. The arrows indicate the area of tumor involvement, located approximately 3.3 cm from the anal verge. Stranding and low signal intensity within the perirectal and mesorectal fat are suggestive of extramural tumor extension. B. Axial T2-weighted pelvic MRI showing a heterogeneous mass within the rectal lumen (circled), consistent with rectal adenocarcinoma. The lesion demonstrates irregular wall thickening with intermediate-to-low T2 signal intensity and suggests involvement of the mesorectal fascia. C. Axial T2-weighted MRI showing extramural extension of the rectal tumor into the mesorectal fat, measuring 20.0 mm in depth. This finding is indicative of advanced T3 stage rectal adenocarcinoma.

A 61-year-old woman presented with a several-month history of constipation and generalized fatigue. Colonoscopy performed on June 22, 2022, revealed an ulcerated lesion on the anterior rectal wall, approximately 3.3 cm from the anal verge. A biopsy was obtained from the lesion (Figure 5). Pelvic magnetic resonance imaging (MRI) showed pathological thickening of the rectal wall measuring 1.7 cm in thickness and extending longitudinally for 8.1 cm (Figure 6). The neoplastic process involved the muscular and serosal layers. In the lower third of the rectum, an endophytic mass was identified within the mesorectal fat, with invasion of the mesorectal fascia and the right levator ani muscle, consistent with extramural invasion. Evidence of EMVI was also present. Multiple mesorectal lymph nodes with irregular shapes and sizes were noted, the largest measuring 12 mm. Additionally, an 8 mm nonhomogeneous lymph node was observed adjacent to the right internal iliac artery. No radiologic evidence of distant metastases was found. Baseline and subsequent CEA measurements remained within normal limits, leading to the classification of this tumor as marker-insensitive with respect to CEA. The tumor was staged as T3N1M0.

From July 5 to September 20, 2022, the patient underwent six cycles of chemotherapy using a modified FOLFIRINOX (mFOLFIRINOX) regimen, consisting of fluorouracil, folinic acid, irinotecan, and oxaliplatin. Follow-up pelvic MRI in October 2022 showed a significant reduction in the size of the primary tumor and affected lymph nodes. Between October 19 and November 22, 2022, the patient underwent radical pelvic radiotherapy, receiving a total dose of 45 gray to the pelvic lymph nodes and rectum, with an additional boost of 5.4 gray to the rectal adenocarcinoma. This was administered in combination with monochemotherapy using capecitabine. Colonoscopy performed on January 25, 2023, demonstrated a complete clinical response (cCR). Given the patient’s preference to avoid surgery, a non-operative approach was pursued following shared decision-making.

Based on a shared decision made during a multidisciplinary tumor board discussion, one additional cycle of capecitabine was administered. Due to a nationwide shortage of capecitabine in Georgia, the regimen was subsequently switched to 5-fluorouracil. Chemotherapy was completed in April 2023.

**Figure 7:**
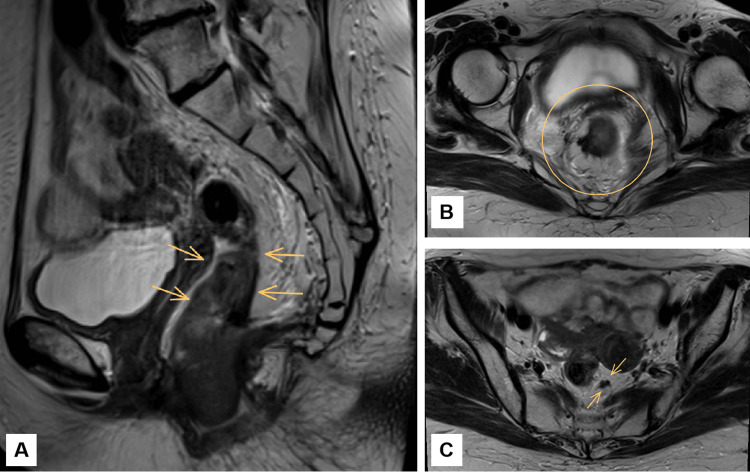
Latest pelvic MRI demonstrating post-treatment findings after total chemoradiotherapy. A. Sagittal T2-weighted pelvic MRI demonstrating uniform hypointense thickening of the rectal wall at the site of the primary tumor (indicated by arrows), consistent with post-treatment fibrosis. No discrete mass or intermediate signal intensity lesion is seen. The mesorectal fat remains preserved, with no evidence of extramural invasion. These findings are indicative of a complete radiologic response. B. Axial T2-weighted pelvic MRI demonstrating concentric thickening of the rectal wall with low signal intensity in the circled area, consistent with post-treatment fibrosis. The mesorectal fascia is preserved, with no evidence of suspicious mesorectal lymphadenopathy. C. Axial T2-weighted pelvic MRI showing fibrotic thickening at the site of the primary tumor (arrows), with no evidence of residual tumor.

A PET/CT scan performed in June 2023 revealed no hypermetabolic activity suggestive of residual or recurrent disease. Colonoscopy during the same period showed post-radiotherapy mucosal scarring, with no evidence of residual malignancy. As of May 2025, the patient remains under active surveillance with no clinical or radiologic evidence of disease recurrence (Figure 7). Surveillance has consisted of pelvic MRI and endoscopic evaluations every four months during the initial two years, followed by every six months thereafter. Chest and abdominal CT scans were obtained at six-month intervals for the first two years and then transitioned to annual imaging.

## Discussion

What makes these two cases noteworthy is that both patients presented with locally advanced rectal adenocarcinoma, achieved a cCR after neoadjuvant therapy, and chose an unconventional management strategy - non-operative "watch and wait" instead of surgical resection. Given the ongoing uncertainty regarding the long-term safety and efficacy of this approach, we believe it is important to share such cases and reflect on their clinical implications.

It is essential to underscore that 20-35% of colorectal cancers have a hereditary component [8], which is why patients are often screened for mismatch repair (MMR) deficiency. The results can significantly impact treatment decisions in certain cases, such as early-stage or metastatic rectal cancers. For example, in dMMR (deficient mismatch repair) tumors, neoadjuvant immunotherapy (e.g., with dostarlimab) has shown promising rates of complete clinical response in early-phase studies [9]. Nonetheless, for most patients, the current NCCN guidelines continue to recommend CRT as the standard of care [10]. In our setting, MMR testing could not be performed due to financial limitations, as immunohistochemistry and microsatellite instability testing are not covered by the national insurance system and are often unaffordable for patients. However, since both of our cases involved Stage III rectal cancer without distant metastases, the absence of MMR status did not meaningfully alter our treatment approach.

Historically, treatment for locally advanced rectal cancer consisted of 25-28 sessions of radiation therapy combined with 5-fluorouracil or capecitabine. More recently, short-course high-dose radiotherapy has emerged as an alternative, offering logistical advantages [6,10]. Still, both NCCN and ESMO guidelines recommend transabdominal surgery following neoadjuvant therapy, and it remains the long-standing standard of care.

However, in 2009, Dr. Angelita Habr-Gama proposed an alternative strategy-"watch and wait"-for patients who demonstrate complete clinical response after neoadjuvant treatment. According to this approach, surgery is deferred in favor of close observation. But how is cCR defined? It includes the absence of detectable tumor on digital rectal examination, endoscopy, and imaging, along with normalization of tumor markers such as CEA. Persistently elevated CEA after treatment suggests incomplete response and the need for further intervention [11].

Several studies have shown that pathological complete response (pCR), the absence of tumor cells in resected specimens, is achieved in 18-26% of cases [12,13]. This observation has led to the consideration of omitting surgery in patients who achieve a complete clinical response after total neoadjuvant therapy (TNT). However, this raises a critical concern: what if we don’t perform surgery, and the tumor regrows? A meta-analysis of 23 studies involving 867 patients reported a two-year local regrowth rate of 15.7%, with 95.4% of these patients successfully treated with salvage therapy [14]. Importantly, overall survival did not differ significantly between patients managed surgically and those under active surveillance. Still, the authors recommended further research to better understand long-term outcomes.

Both of our patients were initially intended to undergo TNT, including induction chemotherapy, chemoradiation, and surgical resection. They had been evaluated and cleared for surgery prior to treatment initiation, and NOM was not part of the original plan. The treatment regimen used was based on NCCN guidelines, which are largely informed by trials such as PRODIGE 23, where TNT was followed by total mesorectal excision (TME) [15]. In our cases, however, surgery was ultimately omitted due to a complete clinical response observed during restaging, and the patients were placed under active surveillance. This represents a case of incidental NOM, where the shift to a non-operative strategy occurs after therapy has begun with curative-intent surgery in mind. This distinction raises an important question: are outcomes equivalent between patients undergoing planned NOM and those managed with incidental NOM? Furthermore, the sequencing of TNT, whether induction or consolidation, is another key variable, with studies like OPRA suggesting higher rates of sustained complete clinical response and organ preservation with consolidation strategies.

Another important consideration is tumor location. Low rectal tumors-typically within 5-6 cm of the anal verge-are more frequently considered for NOM because surgery in these cases often involves APR and permanent colostomy. In contrast, mid- and upper-rectal tumors are less commonly managed non-operatively, and evidence regarding their suitability for watch-and-wait remains less robust. Tumor height, therefore, plays a significant role in patient selection and treatment planning, and should be factored into NOM decision-making.

So how do we justify this strategy, especially when more evidence is needed? Consider that more than one-third of colorectal cancers are stage III, and a significant proportion involve the rectum. Surgical treatment, particularly TME, remains the standard technique but is associated with substantial morbidity (35%), mortality (4-5%), and complications such as urinary and sexual dysfunction, fecal incontinence, and anastomotic leaks [16,17]. Moreover, about 25% of patients face a permanent colostomy. These outcomes can severely affect quality of life-especially in younger patients, among whom rectal cancer incidence is rising. For such individuals, the long-term physical and psychological burden of surgery cannot be underestimated, making organ preservation an important consideration.

Despite its appeal, the "watch and wait" strategy is not without limitations. Accurate assessment of complete clinical response remains challenging. While MRI and endoscopy are highly informative, they may still miss microscopic residual disease, which can lead to tumor regrowth. Thus, patient selection is critical, and stringent follow-up protocols are essential [18,19].

These include physical exams and CEA testing every 3-6 months for the first two years, then every six months for the next three years. Pelvic MRI should be done every six months for the first three years, and CT of the chest and abdomen should be performed every 6-12 months for up to five years. Colonoscopy is recommended one year after treatment, followed by intervals of three years and then every five years, if no abnormalities are found [10].

Clearly, this is a time- and resource-intensive approach that can place a financial and psychological burden on patients. It requires strong patient commitment and robust health system support. Ultimately, the decision to pursue a non-operative strategy must be patient-centered and based on individual values, goals, and risk tolerance. Physicians must be clear about who qualifies for this approach and discuss it within a multidisciplinary team, including oncology, surgery, radiology, and pathology.

Further research is needed to refine the definition of cCR, identify biomarkers or clinical predictors of recurrence, and clarify which patient populations are most suitable for this strategy. Patients, in turn, must be fully informed of both the benefits and risks. For some, the chance to preserve rectal function and avoid life-altering surgery may be worth the rigorous surveillance. For others, the uncertainty and effort involved may outweigh the potential benefits.

## Conclusions

These two cases illustrate that NOM with a watch-and-wait approach can be a viable option for selected patients with locally advanced rectal adenocarcinoma who achieve a complete clinical response after total neoadjuvant therapy. This strategy may offer significant benefits in terms of quality of life, particularly for patients for whom sphincter-sparing surgery is not feasible. However, careful patient selection, shared decision-making, and rigorous follow-up are critical to the success of this approach. While emerging evidence supports its use, further research is needed to refine criteria for complete response and to determine long-term oncologic outcomes compared to standard surgical treatment.
